# MedKnee: A New Deep Learning-Based Software for Automated Prediction of Radiographic Knee Osteoarthritis

**DOI:** 10.3390/diagnostics14100993

**Published:** 2024-05-10

**Authors:** Said Touahema, Imane Zaimi, Nabila Zrira, Mohamed Nabil Ngote, Hassan Doulhousne, Mohsine Aouial

**Affiliations:** 1 MECAtronique Team, CPS2E Laboratory, Ecole Nationale Supérieure des Mines de Rabat, Rabat 10000, Morocco; 2 Ministry of Health and Social Protection, Provincial Ministerial Administration of El Kelaa des Sraghna, El Kelaa des Sraghna 43000, Morocco; 3 Multidisciplinary Research Laboratory for Science, Technology and Society, Department of Computer Engineering and Mathematics, Higher School of Technology, Khenifra, Sultan Moulay Slimane University, Beni Mellal 23000, Morocco; 4 ADOS Team, LISTD Laboratory, Ecole Nationale Supérieure des Mines de Rabat, Rabat 10000, Morocco; 5 Institut Supérieur d’Ingénierie et Technologies de Santé/Faculté de Médecine Abulcasis, Université Internationale Abulcasis des Sciences de la Santé, Rabat 10000, Morocco; 6 Avicenne Military Hospital, Faculty of Medicine and Pharmacy, Marrakech 40000, Morocco; 7 Ministry of Health and Social Protection, Provincial Hospital Center of El Kelaa des Sraghna, El Kelaa des Sraghna 43000, Morocco

**Keywords:** knee osteoarthritis, Kellgren and Lawrence (KL), deep convolutional neural network (DCNN), Osteoarthritis Initiative (OAI), computer-aided diagnosis (CAD)

## Abstract

In computer-aided medical diagnosis, deep learning techniques have shown that it is possible to offer performance similar to that of experienced medical specialists in the diagnosis of knee osteoarthritis. In this study, a new deep learning (DL) software, called “MedKnee” is developed to assist physicians in the diagnosis process of knee osteoarthritis according to the Kellgren and Lawrence (KL) score. To accomplish this task, 5000 knee X-ray images obtained from the Osteoarthritis Initiative public dataset (OAI) were divided into train, valid, and test datasets in a ratio of 7:1:2 with a balanced distribution across each KL grade. The pre-trained Xception model is used for transfer learning and then deployed in a Graphical User Interface (GUI) developed with Tkinter and Python. The suggested software was validated on an external public database, Medical Expert, and compared with a rheumatologist’s diagnosis on a local database, with the involvement of a radiologist for arbitration. The MedKnee achieved an accuracy of 95.36% when tested on Medical Expert-I and 94.94% on Medical Expert-II. In the local dataset, the developed tool and the rheumatologist agreed on 23 images out of 30 images (74%). The MedKnee’s satisfactory performance makes it an effective assistant for doctors in the assessment of knee osteoarthritis.

## 1. Introduction

Knee Osteoarthritis (OA) is one of the most common chronic diseases, and happens when the cartilage in the knee joint breaks down. The resulting friction between the bones of the knee joint can cause discomfort, stiffness, or swelling in the knees. If left untreated, severe osteoarthritis of the knee can result in partial or complete disability. Although there is no known treatment for knee osteoarthritis, if a timely and accurate diagnosis is made there are therapies that can lessen symptoms and halt the disease’s progression. Knee OA affects mainly women, the elderly, and the obese. By 2050, 130 million people worldwide will suffer from knee osteoarthritis [1].

In recent years, Artificial Intelligence (AI) has attracted considerable interest in medical imaging [2,3,4,5,6,7]. Particularly, AI was utilized in the diagnosis of knee osteoarthritis based on MRI and X-ray images [8]. In parallel, Computer-Aided Diagnosis (CAD) is becoming more significant for disease diagnosis, classification, and prediction. The work described in this paper uses X-ray images for automated knee osteoarthritis detection based on a pre-trained deep convolutional neural network (DCNN). The motivation of this study is to develop novel software to assist medical professionals in accurately and speedily diagnosing knee arthritis from X-ray images. To accomplish these goals, a pre-trained Xception model is modified and trained to identify and classify knee osteoarthritis from radiographic images, and then, the automated software, MedKnee, is developed using a graphical user interface (GUI) and the saved Xception model. The model’s performance was analyzed against other CNN models tested on the OAI dataset with comparable sizes. The developed model was validated using an external public database annotated by two experts and a local dataset submitted to a rheumatologist and a radiologist.

## 2. Related Work

In recent literature, numerous researchers have become interested in disease diagnostics utilizing deep learning and machine learning to detect knee OA according to the Kellgren and Lawrence (KL) grading system. Yang et al. [9] developed an automated KOA diagnostic model that can be used in a mobile device called RefineDet. This model consists of two connected models, the Anchor Refinement Module (ARM) used for region of interest (ROI) localization and the Object Detection Module (ODM) for KOA classification. The Transfer Connection Block (TCB) was introduced to share information between the two models. This model was trained and validated on 2579 X-ray images of the posterior-anterior (PA) view of knees collected from the General Hospital of the People’s Liberation Army in China. In this approach, the images were captured using an iPhone held at a distance of 40 cm. The pre-trained network was built on 2499 train images, 263 validation images, and 941 test images. The method achieved an accuracy of 95.7%.

Dalia et al. [10] aimed to detect knee osteoarthritis from radiographic images using VGG16, Resnet-152, and DenseNet-201. The proposed models were developed and compared using 8892 radiographic knee images from the OAI dataset. The ROI was detected using YOLOv5. The best results were obtained using transfer learning of VGG16 by adding two fully connected (FC) layers with 4096 units, a third FC layer with 1000 units, and a softmax activation function. The VGG16 model achieved an accuracy of 69.8%.

Wahyuningrum et al. [11] developed an end-to-end supervised learning model of a Deep Convolutional Neural Network (DCNN) to classify knee OA using three-fold cross validation. The proposed model consists of five convolution blocks. Each block includes a Rectified Linear Unit (ReLU) layer, a max-pooling layer, a flatten layer, a fully connected layer, and a dropout layer. The model achieved an average accuracy of 77.24% when tested using the three folders containing 527, 501, and 528 images.

Yong et al. [12] proposed an Ordinal Regression Module (ORM) to classify knee OA using VGG, ResNet, ResNext, GoogLeNet, DenseNet, and Mobilenet. The proposed ORM splits the probability space of the scalar output into K classes using K-1 cut-points to perform ordinal regression. The neural networks were optimized using a Cumulative Link (CL) loss function and trained using 4130 X-ray images belonging to the OAI dataset. The best result was obtained using DenseNet-161 with an accuracy of 88.09%.

Ruikar et al. [13] developed an automated computer-aided diagnosis, Osteoarthritis network (OACnet), based on a deep neural network for convincing detection of Knee OA. This model was trained on 4746 radiological images acquired from the OAI dataset. It was built from scratch and with hand-crafted feature engineering (joint space narrowing, bone spur, sclerosis, and deformation). The model achieved an accuracy of 83.74%, improved to 92.7% when combined with hand-crafted features.

Wahyuningrum et al. [14] developed a CNN-based system using ReseNet, VGGNet, and DenseNet to diagnose knee OA. The proposed system was implemented using Long-Term Memory (LSTM) as a special kind of CNN that can memorize information and store it in complex network elements over a long period. The proposed system was trained and validated using 5148 X-ray images obtained from the OAI dataset. The LSTM combined with VGG16 achieved the highest accuracy of 75.28%.

Both Chen et al. [15] and Wani et Saini. [16] proposed a novel adjustable ordinal loss instead of the cross-entropy loss in the detection of knee OA using VGG, ResNet, DenseNet, and InceptionV3. To develop and compare the proposed models, 8260 knee X-ray images were used in [15] and 1656 X-ray images were applied in [16], all collected from the OAI dataset. The VGG19 model with the proposed ordinal loss in [15,16] obtained the highest knee severity grading accuracy of 70.4%, and 96.7%, respectively. The ordinal loss function-based approach was used also by Jain et al. [17] to develop an automated method of detecting knee osteoarthritis from X-ray images, named High-Resolution Network (HRNet). The developed model was combined with a convolutional mass attention module (CBAM). HRNet is a revolutionary multi-resolution deep CNN consisting of a convolution (2D) layer followed by layers that add up the high-to-low resolution and then merge the multi-resolution in parallel for information exchange. The model was built on 8260 knee X-ray images from the OAI dataset. The method achieved an accuracy of 71.74% and a mean absolute error (MAE) of 0.311.

Yunus et al. [18] tended to apply a specific approach based on Darknet-53 and Alexnet combined with local binary pattern (LBP) to extract deep features and identify knee OA severity from radiological images. The final classification was performed with the support vector machine (SVM) and the K-nearest neighbors (KNN). Then the classified images were localized using a combination of YOLOv2 and an open neural network exchange (ONNX) built in 24 layers for the preparation of the developed model as (i) input layer, (ii) two element-wise Affine layers, (iii) four convolutional layers, (iv) four Batch normalization (BN) layers, (v) three max-pooling layers, and (vi) four activation layers, while YOLO-v2 was built using three convolutional layers, two BN layers, and two ReLU layers. This approach was developed using 3795 X-ray images from the OAI public dataset. The model achieved an accuracy of 90.6%.

Hu et al. [19] developed a novel deep learning architecture, Adversarial Evolving Neural Network (A-ENN), for the longitudinal progression of Knee OA severity over 4 years. The deep learning model was built with Resnet-18 and three classifiers: VGG19, ResNet50, and visual transformer model (Vit). The proposed model was trained and tested on 3294 labeled knee X-ray images belonging to the OAI dataset. The model combined with VGG19 achieved the best accuracy of 64.6%, 63.9%, 63.2%, 61.8%, and 60.2% for progression baseline, 12-month, 24-month, 36-month, and 48-month, respectively.

Raisuddin et al. [20] proposed and evaluated Deep Active Learning (DAL) designed to classify knee OA severity. The proposed model was built with Semi-Supervised Learning (SSL) deep Siamese using the VGG and Consistency Regularization (CR) approach which ensures the model’s stability in front of the input noise. This model was trained and validated using 8953 knee X-ray images from the OAI dataset. The developed DAL achieved a balanced accuracy of 64.13%.

Huu et al. [21] applied the transfer learning of VGG16 for the automated binary classification of KOA severity using a deep Siamese convolution neural network. The proposed model consists of six convolutional layers with a stride of 1, three convolutional layers with a stride of 2, three dropout layers, a Separable Adaptive Max-pooling (SAM) layer, and a fully connected layer. The proposed model was built using 2874 X-ray images collected from the OAI dataset. The updated VGG16 model achieved an accuracy of 89%.

Yifan et al. [22] presented a knee OA classifier using the Transfer learning of ReseNet34 and DenseNet121 combined with a novel learning scheme that splits data into two categories based on reliability. The two models were developed using 8302 X-ray images from the OAI dataset and a hybrid loss function to manipulate the lower reliability sets. Both models, DenseNet121 and ReseNet34, achieved an accuracy of 70.13% and 68.32%, respectively.

More recently, Alshamrani et al. [23] proposed transfer learning models based on sequential CNNs, VGG16, and ResNet-50 to identify normal and abnormal knees from X-ray images. The proposed models were trained using 3836 X-ray images collected from Kaggle. The best developed model was VGG16 which achieved a training accuracy of 99% and a testing accuracy of 92%.

Mohammed et al. [24] suggested a binary classification and a multiclass classification for the severity of KOA from radiographic images. This approach was built using six pre-trained DNN models: ResNet101, MobileNetV2, VGG16, VGG19, InceptionResNetV2, and DenseNet121. The designed models were trained and tested on 9786 knee images taken from OAI. The best-performing model was the pre-trained ResNet101 for three classes and five classes with an accuracy of 89% and 69%, respectively.

Pi et al. [25] presented an ensemble network based on DenseNet-161, EfficientNet-b5, EfficientNet-V2-s, RegNet-Y-8GF, ResNet-101, ResNext-50-32×4d, Wide-ResNet-50-2, and ShuffleNet-V2-×2-0. The proposed method was implemented using 8260 images from the Osteoarthritis Initiative with optimal image sizes for training the various deep learning models. The optimized model ensemble network achieved an accuracy of 76.93%. Table 1 summarizes the binary and multiclass classification techniques utilized in recent publications based on internal validation.

## 3. Materials and Methods

Although radiography is fast, inexpensive, non-invasive, and easy to use, the quality of the radiographic image requires several treatments to improve contrast and brightness and remove noise. Therefore, before selecting and improving the classification network, image processing was carried out to enhance the identification of knee osteoarthritis severity based on KL grade.

### 3.1. Dataset Description

OAI is a longitudinal observational study conducted by the US National Institutes of Health (NIH) in men and women over ten years. It consists of 4446 X-ray images of knees labeled by Boston University according to the Kellgren and Lawrence (KL) scoring system. In this study, the knee X-ray images used to train the proposed model belong to the OAI dataset available on Mendeley Data [26]. The images consist of unilateral PA fixed flexion of uniform size with an identical resolution. The raw images are bilateral PA fixed flexion with varied resolutions and sizes, pre-processed before being made available for download on Mendeley. The dataset used is labeled Healthy, Doubtful, Minimal, Moderate, and Severe, with equal size to avoid the problem of an unbalanced dataset. The dataset was augmented into 5000 images and then divided into a training set (70%), a validation set (10%), and a testing set (20%).

### 3.2. Preprocessing

**Figure 1:**
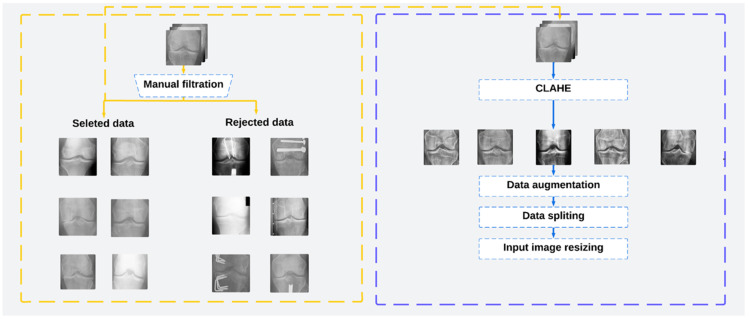
The preprocessing pipeline used in this work.

Histogram equalization is applied in image processing to spread and evenly distribute pixel values. This allows the image quality to be improved by increasing the dispersion of the highest frequency and decreasing the dispersion of other frequencies, allowing the low contrast of the source images to be improved [27]. In this work, we have applied the Contrast Limited Adaptive Histogram Equalization (CLAHE) algorithm used in [21,25]. Then, to improve the performance of the osteoarthritis detection system, it was necessary to use images with variable contrast, brightness, and positions, as they can be captured by the camera used in our work. The original images were resized to 224 × 224, flipped horizontally and vertically, and rotated left and right. Therefore, the image intensity was normalized between 0 and 1, and then the brightness was modified in the range of (0.1,0.7). The preprocessing pipeline is shown in Figure 1.

### 3.3. Network Architecture

Xception, also known as the advanced variant of an Inception module, is a deep convolutional neural network based entirely on depthwise separable convolution layers. It was developed in 2017 by the creator of the Keras Library, Francois Chollet [28]. This model moderately surpassed Inception V3 on the ImageNet dataset in 2017. The pre-trained version of the network trained on more than a million images from the ImageNet database. The pre-trained Xception model was selected in this work because it consumes few resources while maintaining acceptable accuracy, and its architecture is very easy to define and modify, making it a prime candidate for medical tasks [29]. It has been utilized in various medical tasks during the past two years, such as the assessment of benign and malignant gastric ulcer lesions based on gastrointestinal endoscopic images [30], the detection of COVID-19 from radiographic images [31], and the detection of knee osteoarthritis [32].

**Figure 2:**
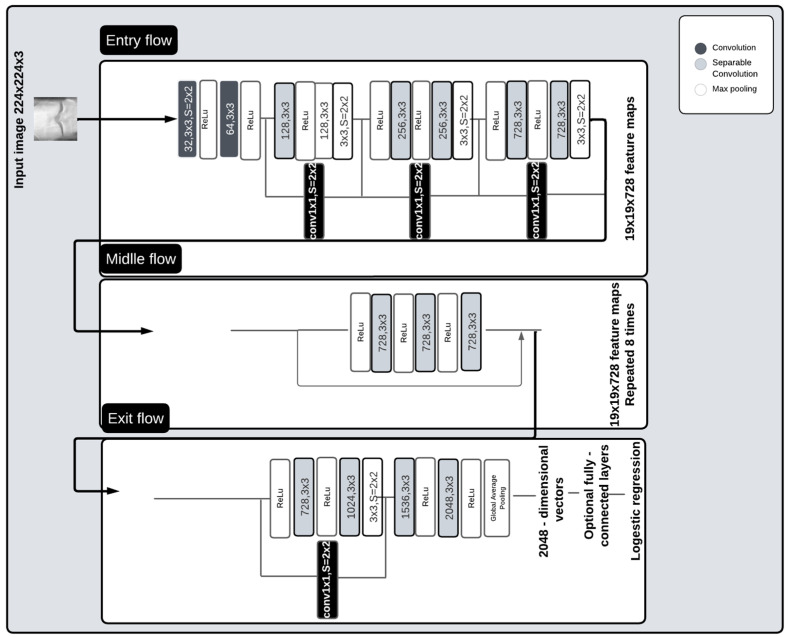
Pre-trained Xception architecture.

The Xception architecture is easy to define and modify. As shown in Figure 2, it contains 2 convolutional layers, 34 separable convolution layers, 4 max-pooling layers, and a global average pooling layer. Convolutional and separable convolution layers are followed by batch normalization and ReLU layers. The 36 convolutional layers used for extracting network characteristics are arranged in 14 blocks, which are all surrounded by linear residual connections, except for the first and last layers. The convolutional base is succeeded by a logistic regression layer.

The pre-trained model head in this study has been updated with a global average pooling layer, batch normalization layer, a dropout of 0.2, and a fully connected layer with a softmax activation function. The model is trained using categorical cross-entropy with five outputs corresponding to the Kellgreen and Lawrence (KL) grading scale. The updated network was trained for 200 epochs using a batch size of 16, and an Adam optimizer with an initial learning rate of 0.0001. The best model was saved in a «.H5» file. The network training is conducted with the hyperparameters illustrated in Table 2.

### 3.4. Software Requirements and Libraries

To carry out this work, a machine running the Windows 10 operating system with an i7 processor, 16 GB memory, and a Nvidia Quadro M2200 5.2 graphics processing unit (GPU) is used. Python 3.9.16 (Anaconda3), TensorFlow-GPU 2.10.0, CUDA (Compute Unified Data Architecture) 11.2, and CUDNN 8.1.33 are used to implement the proposed method. Spyder5 was used in these experiments as a framework.

## 4. Experiment Results and Model Deployment

The saved model was deployed in a graphical user interface (GUI) using a desktop application. Then, to validate our study, the model was tested on the OAI labeled dataset and two other external datasets, including a local dataset.

### 4.1. Result on OAI Dataset

The proposed automatic system’s performance was assessed through the use of the F1-score, precision, recall, and confusion matrix. The pre-trained model is tested on 1000 images belonging to the OAI dataset, divided into 200 images for each KL grade. The use of batch normalization and drop-out layer has enabled us to avoid overfitting. The model achieved a validation accuracy of 99.39% and a test accuracy of 97.20%.

**Figure 3:**
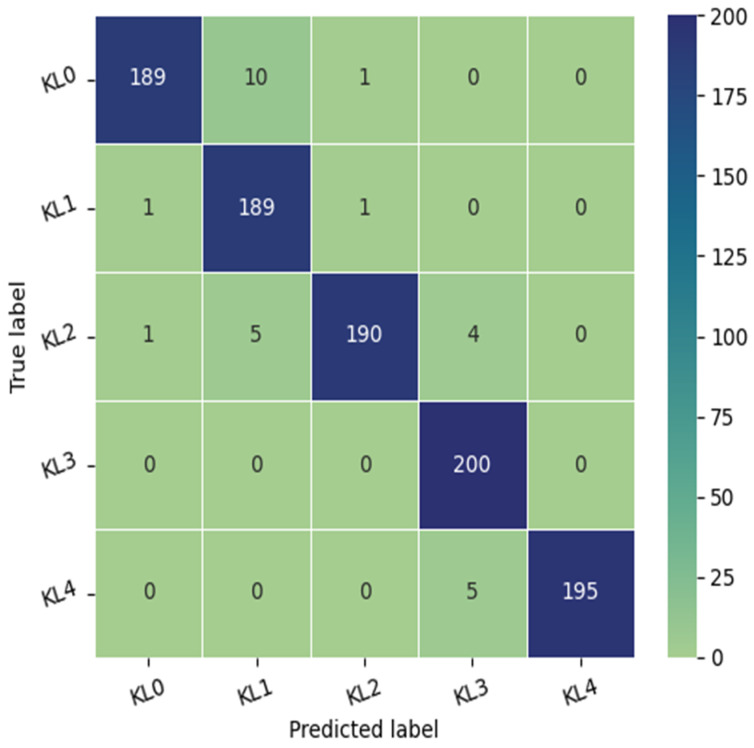
The confusion matrix of the proposed approach tested on the OAI dataset.

The test dataset consisted of 1000 knees, 800 knees with OA and 200 knees without OA. As illustrated in Figure 3, the confusion matrix of the Xception model indicates that KL3 was classified without error, followed by KL grade 1, where the method correctly classified 198 knees out of 200. In third place, we find KL4 grade with a re-rating rate of 195 correctly identified knees. Finally, KL2 and KL0 grades have the lowest rate with the correct identification of 190 and 189 knees, respectively. We note the classification of grade KL0 as grade KL 1 in 10 cases due to the minimal difference between the two grades and the difficulty in distinguishing between them.

As shown in Table 3, the model perfectly detects grade KL3 with a Recall of 1 and an F1-score of 0.98. KL1 and KL4 were classified with a recall of 0.99 and 0.97, respectively. KL0 and KL2 were identified with an equal precision of 0.99 and an F1-score of 0.97.

Table 4, compares the proposed model with works deployed on an identical dataset collected from the OAI dataset with a comparable size. The proposed model has outperformed many research studies based on internal validation with a validation accuracy of 99.39% and a test accuracy of 97.20%.

### 4.2. Graphical User Interface (GUI): MedKnee

**Figure 4:**
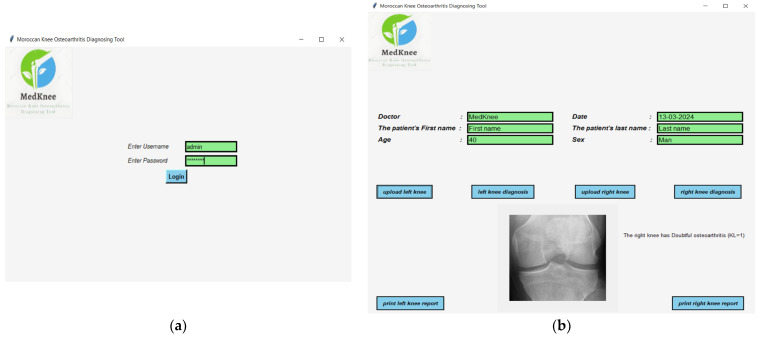
(**a**) Login interface; (**b**) Diagnostic result interface.

A desktop application, the Moroccan KOA diagnosing tool (MedKnee), has been created using TKINTER and FPDF2 Python to simplify the use of the knee osteoarthritis classification implementation. The interface allows the user to enter the patient’s name, date, age, and sex. After entering the correct password, the user can select the left or right knee and obtain an individual diagnosis for each knee (Figure 4).

**Figure 5:**
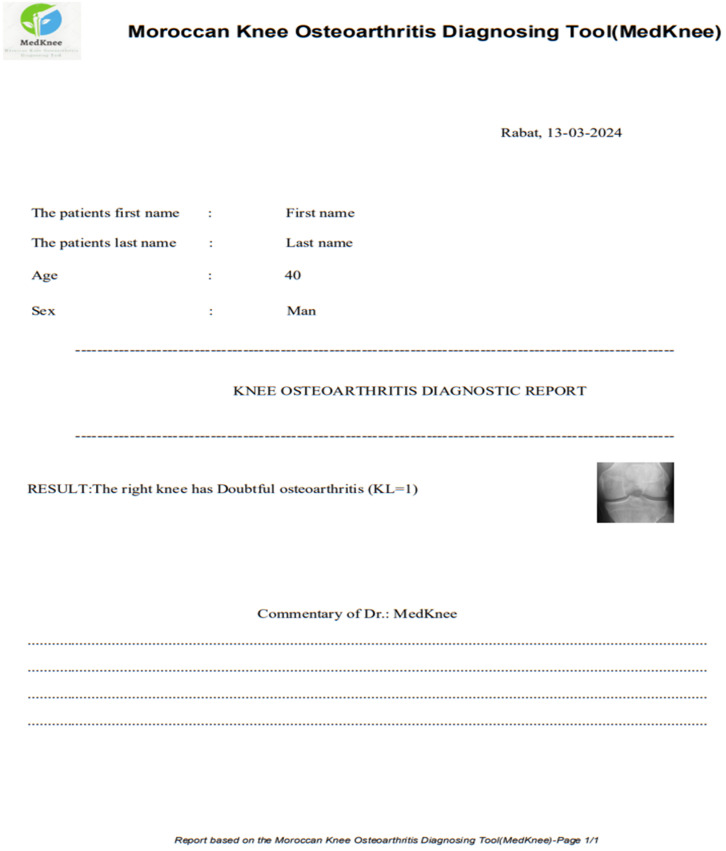
Printable report on knee osteoarthritis diagnosis.

As shown in Figure 5, the diagnostic report for each knee can be printed, with a field at the bottom for comments.

### 4.3. Results on External Validation

In the first step, to approve our study, Medical Expert-I and Medical Expert-II public datasets were used. Then, a local dataset was employed in the second step to compare the results with a specialist’s diagnosis.

#### 4.3.1. Medical Expert Public Dataset

**Figure 6:**
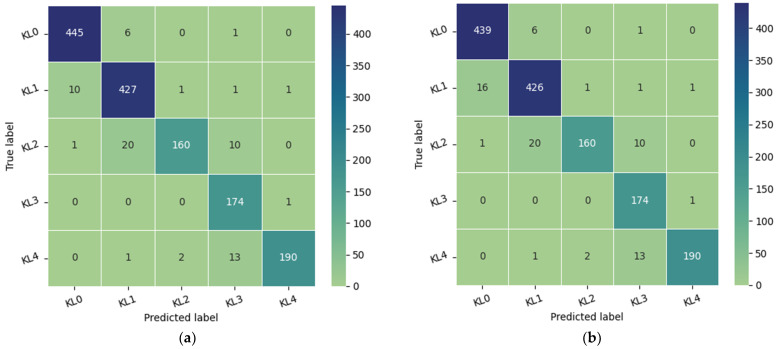
Model performance: (**a**) the confusion matrix of the model tested in 1464 images of Medical Expert-I; (**b**) the confusion matrix of the proposed approach tested on 1463 images of Medical Expert-II.

The database is composed of 1650 digital radiographs of the knee joint collected from various hospitals and diagnostic centers in India [33]. Each X-ray image of the knee is manually labeled by two medical experts according to the Kellgren and Lawrence grades. Both experts are experienced orthopedic surgeons who review between 70 and 100 radiographs per day. To obtain images comparable to those in the Medknee training dataset, preprocessing consistent with that applied to the aforementioned training dataset must be implemented. First, we excluded images with double knees, then we resized the selected images from 362 × 162 to 224 × 162, and finally, we applied contrast-limited adaptive histogram equalization (CLAHE) to improve the local contrast of the images. As shown in Figure 6, the confusion matrix was used to evaluate the performance of our model. The proposed model achieved an accuracy of 95.36% on 1464 images labeled by expert1 and 94.94% on 1463 images annotated by expert2.

#### 4.3.2. Local Dataset

**Figure 7:**
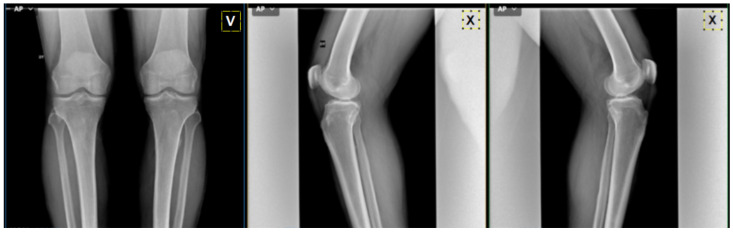
Example of knee X-ray images collected and filtered from patients with knee symptoms.

**Figure 8:**
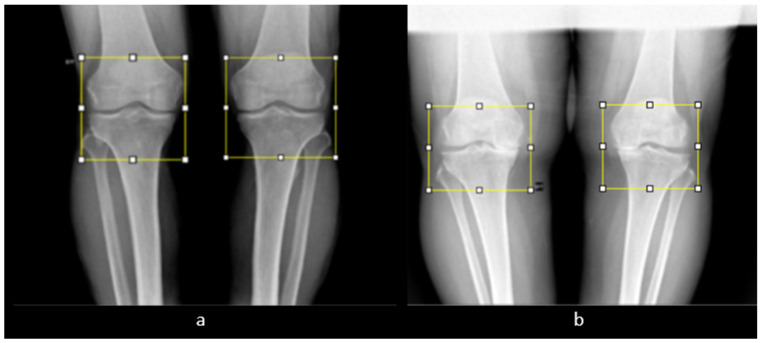
Manual ROI selection. (**a**) ROI selection of a man’s knee. (**b**) ROI selection of a woman’s knee.

In the local dataset, we have selected 60 adult patient files of men and women with knee disorders who were radiographically examined at the Radiology Department of the El Kelaa des Sraghna Provincial Hospital. Each file consists of one or more knee radiographs in Dicom format as presented in Figure 7. The images were acquired using a standard ITALRAY radiology table. After analysis, 30 images with implants and non-posterior-anterior examination views were excluded and 30 left and right Dicom knees were retained to validate this work. To make the validation local dataset, the ROI was selected manually as illustrated in Figure 8. Then, each patient’s file was presented to a rheumatologist with PNG images. In case of disagreement between our diagnostic system and the rheumatologist, a radiologist was consulted for arbitration to make the final decision.

Out of the 30 images presented to the doctor, the DL model and the rheumatologist agreed on 23 images (74%). As shown in Table 5, in the seven remaining images where there was a disagreement, the referee confirmed four images, indicating agreement between the referee and the DL model. Three images were not confirmed, of which only one was not confirmed by the referee, indicating agreement between the referee and the rheumatologist. Based on the final decision of the arbitrator physician, our diagnostic model failed in 2 out of 30 images with a test accuracy of 90%.

## 5. Discussion

In this work, an automated approach to classify the severity of knee osteoarthritis from simple radiographic images according to the KL grade is presented and implemented in a desktop application using TKINTER and the DL model based on Xception. The pre-trained Xception was chosen due to its superior performance on the OAI dataset compared to other models used during software development, such as EfficientNetV2M (93.15%) and MobileNetV2 (75.60%). Table 4 gives the multiclass classification values in comparison with similar works. The developed method achieved a validation multiclass accuracy of 99.39% and a test accuracy of 97.20%, which is a high performance compared to [11,12,14]. To validate our work, we tested the model on the Medical expert database and a local database. The model achieved an accuracy of 95.20% on Medical Expert-I and 94.94% on Medical Expert-II. This minimal difference confirms the results obtained in [34]. To validate the results, the model was tested on 30 images and compared with the diagnosis of a rheumatologist. In case of disagreement, a radiologist was consulted. Out of the 30 images, 28 were correctly identified (90%). It is worth noting that when four images were presented twice to the same doctor, the latter failed to give the same diagnosis for two images. However, our software’s diagnosis coincided with the second diagnosis of the doctor for one of the images, which was then revised. Furthermore, it is often challenging to differentiate between images of grades KL-0 and KL-1, which explains the discrepancy in identifying these two grades, but there is no clinical benefit in distinguishing between KL grades 0 and 1. In binary classification for osteoarthritis (OA) (KL < 2), and non-OA (KL ≥ 2), the new software MedKnee tested on a local dataset achieved an accuracy of 100%. Indeed, as shown in Table 5, although only one knee was classified as doubtful (KL = 1) by the rheumatologist, the referee (radiologist) confirmed the software’s diagnosis by validating that the knee in question had minimal osteoarthritis (KL = 2). In 2023, similar software, MediAI-OA [35], was developed using the NASNet DL model, but its accuracy is limited to 83%, which is significantly lower than the performance of our new software, MedKnee.

However, several limitations must be noted regarding the proposed approach. First, the study of the OAI dataset did not include lateral radiographs. Therefore, as noted by Ahmed and Mustapha [1], the addition of lateral radiographs would have provided additional information. Secondly, the radiographic images of knee osteoarthritis utilized to train the proposed model are pre-processed and consist of a PA radiograph with fixed flexion and identical resolution and size, whereas raw images require pre-processing before they can be processed. The generalizability of the developed model to external databases is another major limitation. The proposed model is limited to pre-processed images with zooming, resizing, and region of interest (ROI) rearrangement. Since the model was only developed using the pre-treated images from the OAI dataset, its accuracy is not acceptable in the absence of these operations. Nonetheless, the model can be more broadly generalized without requiring significant preprocessing if it is built using a combination of many external databases and local or other institutional datasets, including bilateral and unilateral images of varying sizes and resolutions. Furthermore, the developed application helps physicians to identify knee osteoarthritis with acceptable accuracy using manual localization of the (ROI). The addition of deep learning models, such as YOLO or Faster-RCNN, would be helpful for real-time detection of the ROI. Finally, the implementation of the proposed approach in this study with enhanced computational resources, including Nvidia GeForce, can enhance the degree of accuracy achieved by increasing the number of training epochs and images. In the future, we expect to improve our application by using a large local dataset and introducing automatic ROI selection with the option of adding image segmentation. Nevertheless, the developed software achieved a high level of accuracy and can help physicians predict the exact severity grade of knee OA by analyzing radiographic images.

## 6. Conclusions and Future Work

This work presents MedKnee, new DL software designed to automatically classify the severity of knee osteoarthritis from radiographic images. The software is developed based on the transfer learning of a pre-trained Xception model and the public OAI dataset. The best DL model achieved a validation accuracy of 99.39% and a test accuracy of 97.20%, moderately better than reported in the recent literature. The developed tool was validated using the Medical expert dataset and a local dataset. The model performed with an accuracy of 95.36% when tested on Medical Expert-I, and 94.94% on Medical Expert-II. On the local dataset, the model was tested on 30 images, and compared with a rheumatologist’s diagnosis. To resolve the disagreement, a radiologist was consulted. The model achieved a multiclass accuracy of 90% and a binary classification accuracy of 100%. It can be concluded that the proposed software “MedKnee” can equip radiologists with the ability to quickly and accurately diagnose and predict knee osteoarthritis from X-ray images. The proposed tool in this study has the potential to be valuable for the early detection and diagnosis of knee osteoarthritis. Future work on this research could include improving the system’s reliability using a large dataset, as well as automatic ROI segmentation and localization. In addition, the deployment of the software in Raspberry Pi can be seen as another advantage for offline diagnostics without the use of a traditional computer.

## Figures and Tables

**Table 1:** Overview of the methods used in recent literature with the average values of the metrics used.

References	Year	Classes(n)	Metrics
Yang et al. [9]	2022	5	Accuracy: 95.7%, Recall: 0.975, F1: 0.905
Dalia et al. [10]	2020	5	Accuracy: 69.8%, Recall: 0.674, F1: 0.666
Wahyuningrum et al. [11]	2020	5	Accuracy: 77.24%, Loss: 0.15
Yong et al. [12]	2021	5	Accuracy: 88.09%, MAE: 0.330, QWK: 0.86
Ruikar et al. [13]	2022	5	Accuracy: 92.7%, Recall: 0.92, F1: 0.91
Wahyuningrum et al. [14]	2019	5	Accuracy: 75.28%, Mean loss: 0.09
Chen et al. [15]	2019	5	Accuracy: 70.4%, MAE: 0.358
Wani and Saini [16]	2022	5	Accuracy: 96.7%, MAE: 0.344
Jain et al. [17]	2021	5	Accuracy: 71.74%, MAE: 0.311, QWK: 0.8690
Yunus et al. [18]	2022	5	Accuracy: 90.6%, F1: 0.88, Precision: 0.85
Raisuddin et al. [20]	2022	5	Accuracy: 64.13%
Huu et al. [21]	2022	2	Accuracy: 89%, Loss: 0.29
Yifan et al. [22]	2022	5	Accuracy: 70.13%, MCC: 0.5864
Alshamrani et al. [23]	2023	2	Accuracy: 92%, Recall 0.86, F1: 0.89
Mohammed et al. [24]	2023	3	Accuracy: 89%, Recall: 0.86, F1: 0.86
Pi et al. [25]	2023	5	Accuracy: 76.93%, Recall: 0.7525, F1: 0.7665

**Table 2:** Hyperparameters maintained during training.

Parameter	Value
Input size	224 × 224 × 3
Batch size	16
Learning rate	0.0001
Optimizer	Adam
Epochs	200
Loss function	Categorical cross-entropy

**Table 3:** Performance of the proposed model in detecting each KL grade of knee osteoarthritis validated on the OAI dataset.

Metric	Precision	Recall	F1-Score	Test Data
KL0	0.99	0.94	0.97	200
KL1	0.93	0.99	0.96	200
KL2	0.99	0.95	0.97	200
KL3	0.96	1.00	0.98	200
KL4	1.00	0.97	0.99	200
Macro-average	0.97	0.97	0.97	1000
Mean Accuracy			97.20%	1000

**Table 4:** Comparison between the proposed tool, MedKnee, and similar works in KOA’s 5-class classification based on internal validation.

References	OAI Dataset (Test)	Methods	Accuracy (%)
Dalia et al. [10]	8892 (20%)	VGG16	69.8
Wahyuningrum et al. [11]	4737(521,527,528)	Deep CNN	77.24
Yong et al. [12]	4130 (20%)	DenseNet161+ORM	88.09
Ruikar et al. [13]	9492(1898)	OACnet-Handcrafted features	92.7
Wahyuningrum et al. [14]	5148(479)	VGG16+LSTM	75.28
Chen et al. [15]	8260 (20%)	VGG19-Ordinal	70.4
Wani et Saini. [16]	1656 (20%)	VGG19	96.7
Jain et al. [17]	8260 (1656)	HRNet+CBAM	71.74
Yunus et al. [18]	3795 (1656)	Fine KNN	90.6
Raisuddin et al. [20]	8953 (903)	Deep semi-supervised active learning	64.13
Yifan et al. [22]	8302 (10%)	DenseNet121	70.13
Mohammed et al. [24]	9786(20%)	ResNet101	69
Pi et al. [25]	8260 (1656)	Ensemble Network	76.93
**Ours**	5000 (20%)	MedKnee	97.20

**Table 5:** Arbitration by radiologists to resolve disagreements between software and rheumatologists regarding the local dataset. The bold characters that show that there is an agreement between the result of the developed software diagnostic and the doctors’ diagnostic.

Knee image	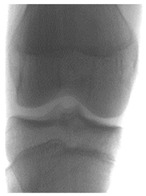	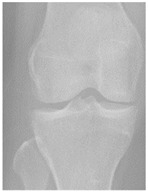	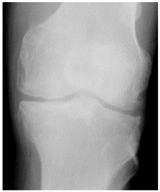	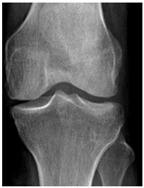	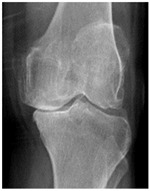	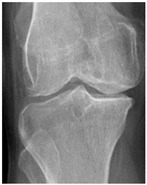	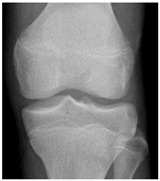
MedKnee	**KL-0**	**KL-3**	**KL-3**	**KL-2**	**KL-3**	KL-4	KL-0
Rheumatologist	KL-1	KL-2/**KL-3**	KL-4	KL-1	KL-4	KL-3	**KL-1**
Radiologist	**KL-0**	KL-1	**KL-3**	**KL-2**	**KL-3**	KL-2	**KL-1**

## Data Availability

A local dataset was constructed to validate the software, However, it is not currently available due to ethical and privacy restrictions. The software was additionally validated using a public dataset called Medical Expert: https://www.kaggle.com/datasets/tommyngx/digital-knee-xray?resource=download (accessed on 25 September 2023).

## Abbreviations

CNN	Convolutional neural network
CAD	Computer-aided diagnosis
DCNN	Deep convolutional neural network
DL	Deep Learning
KL	Kellgren and Lawrence
OA	Osteoarthritis
KOA	Knee Osteoarthritis
OAI	Osteoarthritis initiative
ReLU	Linear rectified unit
ROI	Region of interest
A-ENN	Adversarial evolving neural network
DAL	Deep Active learning
LSTM	Long Short-Term Memory
ORM	Ordinal regression module
HRNet	High-resolution network
CBAM	Convolutional mass attention module
MCC	Matthews correlation coefficients
QWK	Quadratic weighted Cohen’s kappa coefficient
